# Defining and Understanding Treatment Resistance in Obsessive-Compulsive Disorder

**DOI:** 10.1192/j.eurpsy.2023.102

**Published:** 2023-07-19

**Authors:** B. Dell’Osso

**Affiliations:** ^1^Department of Mental Health, Department of Biomedical and Clinical Sciences Luigi Sacco; ^2^“Aldo Ravelli” Center for Neurotechnology and Brain Therapeutic, University of Milan, Milan, Italy; ^3^Department of Psychiatry and Behavioral Sciences, Bipolar Disorders Clinic, Stanford University, California, United States

## Abstract

**Abstract:**

Previously considered a rare condition, OCD is now recognized as a common psychiatric disorder, with lifetime prevalence estimates ranging between 2% to 5%. Rates of resistance to first-line OCD treatments have been reported to be as high as 60%. Several clinical, biological and genetic factors have been investigated as treatment response moderators in OCD. These have included age at OCD onset, symptom subtypes, comorbidity patterns, gender and pharmacogenomics. This presentation will explore the definitions of treatment resistance in OCD as well as what is known about the epidemiology and clinical correlates associated with treatment resistance.

**Disclosure of Interest:**

B. Dell’osso Grant / Research support from: LivaNova, Inc., Angelini, and Lundbeck

